# Importance of Angina Pectoris by Coronary Artery Disease Severity

**DOI:** 10.1001/jamanetworkopen.2026.43292

**Published:** 2026-11-11

**Authors:** Kerstin Welén Schef, Christian Dworeck, Nina Johnston, Sofia Sederholm Laweson, Stefan Söderberg, Per Tornvall, Moman Aladdin Mohammad, Tomas Jernberg

**Affiliations:** 1 Department of Clinical Sciences, Danderyd Hospital, Karolinska Institutet, Stockholm, Sweden; 2 Department of Cardiology, Danderyd University Hospital, Stockholm, Sweden; 3 Department of Molecular and Clinical Medicine, University of Gothenburg Sahlgrenska Academy, Gothenburg, Sweden; 4 Department of Cardiology, Sahlgrenska University Hospital, Gothenburg, Sweden; 5 Department of Medical Science, Cardiology, Uppsala University, Uppsala, Sweden; 6 Department of Health, Medicine, and Caring Sciences, Linköping University, Linköping, Sweden; 7 Clinical Department of Cardiology, Region Östergötland, Linköping, Sweden; 8 Department of Public Health and Clinical Medicine, Heart Centre, Umeå University, Umeå, Sweden; 9 Department of Clinical Science and Education, Karolinska Institutet, Södersjukhuset, Stockholm, Sweden; 10 Division of Cardiology, Department of Clinical Sciences Lund, Lund University, Lund, Sweden; 11 Department of Cardiology, Skåne University Hospital, Lund, Sweden

## Abstract

**Importance:**

Angina pectoris remains a common clinical manifestation, but contemporary population-based data on its long-term prognostic significance are limited.

**Objective:**

To evaluate the long-term importance of angina pectoris in association with coronary artery disease (CAD) severity determined by coronary computed tomography angiography (CCTA) in a general population.

**Design, Setting, and Participants:**

This population-based cohort study included participants from the Swedish Cardiopulmonary Bioimage Study who had completed the Rose Questionnaire and underwent valid CCTA from November 2013 to October 2018, excluding participants with prior myocardial infarction (MI), heart failure, or coronary revascularization. Follow-up continued through September 2024. Data analyses were conducted between August 2025 and January 2026.

**Exposures:**

Angina (defined by the Rose Questionnaire) and CAD (categorized as no findings, nonobstructive CAD [<50% stenosis], or obstructive CAD [≥50% stenosis]) according to CCTA findings.

**Main Outcomes and Measures:**

The primary outcome was MI or coronary death. The secondary outcome was major adverse cardiac events (MACE), defined as MI, coronary revascularization, heart failure hospitalization, or death from coronary disease or heart failure. Associations were evaluated using Cox regression with sequential multivariable adjustment and stratification by CAD category and presented as hazard ratios (HRs).

**Results:**

This study included 24 176 individuals (median [IQR] age, 57 [54-61] years; 12 225 female [50.6%]; 1615 with hyperlipidemia [6.8%]; 4374 with hypertension [18.3%]; 703 with diabetes [2.9%]). Angina was present in 720 participants (3.0%). During a median (IQR) follow-up of 7.7 (6.9-8.6) years, 293 primary events occurred. Angina was associated with a higher incidence of MI or coronary death compared with no angina (21 participants [2.9%] vs 272 participants [1.2%]; HR, 2.50; 95% CI, 1.60-3.90). Event rates increased across CAD categories, with no evidence of interaction between angina and CAD severity. After multivariable adjustment, angina remained independently associated with the primary outcome (HR, 1.65; 95% CI, 1.04-2.63). Similar associations were observed for MACE (adjusted HR, 2.34; 95% CI, 1.73-3.16).

**Conclusions and Relevance:**

In this cohort study of a contemporary general population, angina pectoris was associated with adverse cardiac outcomes, independent of CAD severity assessed by CCTA. These findings suggest that symptom assessment remains relevant in cardiovascular risk stratification because it provided incremental prognostic information that may reflect underlying coronary microvascular dysfunction or vasomotor disorders.

## Key Points

**Question:**

What is the importance of angina pectoris in a general population in association with coronary artery disease severity?

**Findings:**

In this cohort study of 24 176 middle-aged individuals, angina pectoris was associated with an increased risk of myocardial infarction and coronary death across all categories of coronary artery disease after multivariable adjustment.

**Meaning:**

These findings suggest that angina pectoris identifies elevated cardiovascular risk beyond coronary epicardial atherosclerosis and should be integrated with coronary imaging findings and clinical background factors in contemporary risk assessment.

## Teaser

This cohort study evaluates the long-term associations of angina pectoris with adverse cardiac outcomes overall and by coronary artery disease severity in a general population of Swedish adults.

## Introduction

Expanding knowledge of the complexity of coronary artery disease (CAD) has led to updates of definitions of its stable form, chronic coronary syndrome. Coronary microvascular dysfunction (CMD) and other functional coronary disturbances are now recognized as important pathogenetic mechanisms, broadening the spectrum of clinical presentations of chronic coronary syndrome, in which angina pectoris remains common. Prognostication of patients with chronic coronary syndrome may therefore be challenging.^1^

Studies evaluating the association of angina pectoris with cardiovascular events are largely noncontemporary.^2,3,4,5,6,7,8,9^ Over the past decades, research has instead focused on the prognostic importance of CAD severity among patients with angina.^10,11,12^ Systematic reviews have reported increasing annual cardiac event rates with greater CAD severity, with very low absolute risks among patients with angina and normal coronary arteries.^13,14^ Plaque burden and adverse plaque characteristics have also been identified as predictors of nonfatal and fatal myocardial infarction (MI).^12,15,16^ Fewer studies have compared the prognosis of CAD among patients with angina with population-based controls without angina, but available data suggest similar patterns.^17^ To date, no study has examined the association of angina pectoris with subsequent cardiac events stratified by coronary anatomy in a random sample from a general population.

Previously published data from the Swedish Cardiopulmonary Bioimage Study (SCAPIS) have reported both a high prevalence of silent coronary atherosclerosis, detected by coronary computed tomography angiography (CCTA), and significant prevalence of angina pectoris with a low magnitude of association with obstructive CAD (≥50% stenosis).^18,19^ Therefore, by investigating long-term data from SCAPIS, the present study aimed to evaluate the prognostic value of angina pectoris in a contemporary general population of middle-aged individuals without established cardiac disease and associations with CAD severity as assessed by CCTA.

## Methods

### Study Design and Population

This cohort study was approved by the Swedish Ethical Review Authority and was conducted in accordance with the principles of the Declaration of Helsinki.^20^ Reporting followed the Strengthening the Reporting of Observational Studies in Epidemiology (STROBE) reporting guideline. The source population of this cohort study consisted of participants from the SCAPIS study, which included 30 154 randomly selected individuals from the general population, aged 50 to 64 years, recruited at 6 university hospital sites in Sweden from November 2013 to October 2018. The SCAPIS study protocol, including details of the recruitment process and population, has been published elsewhere.^21^ In summary, the Swedish population register was used to randomly select individuals for study invitation, with the only exclusion criteria being a lack of Swedish personal identification number or inability to understand written or spoken Swedish for informed consent. All SCAPIS participants completed an extensive study questionnaire at baseline, including questions from the Rose Angina Questionnaire,^22^ and most underwent CCTA (90.3%).

The present study population comprised SCAPIS participants who had completed the Rose Angina Questionnaire and undergone a valid CCTA, consistent with our published cross-sectional study.^18^ Participants with a previous diagnosis of MI, heart failure, or previous coronary revascularization were excluded. eTable 1 in Supplement 1 provides relevant *International Statistical Classification of Diseases and Related Health Problems, Tenth Revision (ICD-10)* codes and codes for classification of in-patient care-related procedures (KVA, the Swedish procedural coding system) for the exclusion criteria. Study participants were followed up from the date of completion of CCTA until September 30, 2024, or until antecedent emigration or death. Written informed consent was obtained from all participants.

### Angina

The Rose Angina Questionnaire includes 4 standardized questions on exertional chest pain and symptom relief; individuals reporting exertional chest pain are further classified, according to symptom behavior and duration, as probable or definite angina (eFigure 1 in Supplement 1). In this study, angina categorization included both probable and definite angina, while individuals without exertional chest pain were categorized as no angina in accordance with previous prognostic studies and to maintain consistency with our previous analysis of angina prevalence and coronary atherosclerosis in the same cohort.^18,23,24,25^ The original Rose Angina Questionnaire also includes a site map for chest pain localization, intended to be filled in by hand.^22^ However, this could not be implemented technically in the SCAPIS questionnaire and was hence not used in the present study.

### CCTA Findings

Details of the CCTA procedure in SCAPIS and how images were read can be found in previous publications.^21^ In the present study, we defined a valid CCTA as having assessable proximal segments (1, 5, 6, and 11), without technical failure. According to the degree of coronary atherosclerosis in the 18 segments, individuals were categorized as follows: (1) no findings (no coronary atherosclerosis in all segments), (2) nonobstructive CAD (<50% stenosis or not assessable due to calcium artifacts in any segment), and (3) obstructive CAD (≥50% stenosis in any segment). Total plaque burden was also evaluated in SCAPIS, using the segment involvement score (SIS), ranging from 0 to 16, with a higher score indicating a greater extent of coronary plaque burden.

### Baseline Characteristics

All background variables included in the study were obtained through the SCAPIS baseline examination and associated questionnaire, which covered questions on sociodemographic background, lifestyle habits, previous or current diseases, as well as psychology.^21^ Estimated glomerular filtration rate was calculated using the Chronic Kidney Disease Epidemiology Collaboration formula, and chronic kidney disease was defined as an estimated glomerular filtration rate less than 60 mL/min/1.73m^2^.

### Outcomes

Outcome data were obtained through the Swedish National Patient Registry and Cause of Death Register. The primary end point for this study was MI or death of coronary cause. The secondary end point was a composite of MI, revascularization, heart failure hospitalization, death of coronary cause, or heart failure death (ie, major adverse cardiac events [MACE]), whichever occurred first. Heart failure hospitalization or heart failure death was examined as a tertiary end point. Relevant *ICD-10* and KVA codes for the outcomes are provided in eTable 1 in Supplement 1.

### Statistical Analysis

Data analyses were conducted between August 2025 and January 2026. Statistics were presented as frequency and percentage for categorical variables and mean (SD) or median (IQR) for continuous variables, depending on variable distributions. For background statistics, we did not perform any hypothesis testing because associations have been published previously.^18^ To describe the frequency of events according to time since CCTA, we constructed Kaplan-Meier curves, and the results of the rate of events were analyzed using a log-rank test. Univariable and multivariable Cox proportional hazards models were constructed to compare risk between groups for the primary, secondary, and tertiary end points. The main analysis included all participants with angina compared with all participants with no angina, followed by 3 stratified analyses according to CCTA outcome: (1) angina compared with no angina among participants with no findings, (2) angina compared with no angina among participants with nonobstructive CAD, and (3) angina compared with no angina among participants with obstructive CAD. The possible interaction between angina and CAD severity was evaluated using Cox regression, including the interaction term.

Proportional hazards assumptions were assessed using visual inspection of Kaplan-Meier plots and Schoenfeld residuals, with no evidence of violation. A hazard ratio (HR) and 95% CI were calculated from the Cox regression models.

We used directed acyclic graphs to visualize the potential associations of background factors with exposures and outcomes (eFigure 2 in Supplement 1). Two multivariable Cox regression models for the primary and secondary end points were used: (1) a partially adjusted model including all 19 included covariates (sociodemographic, lifestyle, and psychological factors, as well as current or previous comorbidities) and (2) a fully adjusted model adding the CAD severity category (only applicable for the main analysis) and SIS (not applicable in the stratified analysis of participants with no findings). Continuous variables were categorized prior to analysis and entered into the Cox regression model as categorical variables, using the lowest category as reference group. To minimize overfitting, multicollinearity was assessed using variance inflation factors. The multivariable model used for the tertiary end point was limited to age and sex due to the low number of events.

As sensitivity analyses, 2 additional adjustment models were employed: (1) incorporating self-reported (SCAPIS questionnaire) histories of MI, coronary revascularization, or heart failure into the main models and (2) additionally including the presence of aortic valve calcification at CCTA, to account for possible angina related to aortic valve stenosis. Missing data on background variables were overall low, although considered significant to the survival analyses (eTable 2 in Supplement 1). Based on the observed patterns, missing data were assumed to be missing at random and multiple imputation was performed using the Markov Chain Monte Carlo method with 5 imputations to account for uncertainty in the missing values. The imputation model included all variables used in the adjusted analyses to preserve associations among them. Analyses were conducted on the imputed datasets and pooled according to the Rubin rules.^26^

For all analyses, a 2-tailed *P* < .05 was considered statistically significant. SPSS Statistics version 28 (IBM) was used for data handling, variable coding, and statistical analyses.

## Results

### Study cohort

As demonstrated in the study cohort flow chart (Figure 1), a total of 30 154 individuals participated in SCAPIS, of whom 28 974 had completed the Rose Angina Questionnaire. Of these, 26 325 completed CCTA, of which 1723 had nonassessable proximal segments. Hence, 24 602 individuals had a valid CCTA and were thus eligible for the present study, of which 426 were excluded based on study exclusion criteria. After exclusions, the study population comprised 24 176 individuals (median [IQR] age, 57 [54-61] years; 12 225 female [50.6%]; 1615 with hyperlipidemia [6.8%]; 4374 with hypertension [18.3%]; 703 with diabetes [2.9%]) from the original SCAPIS cohort (eTable 3 in Supplement 1).

**Figure 1.**
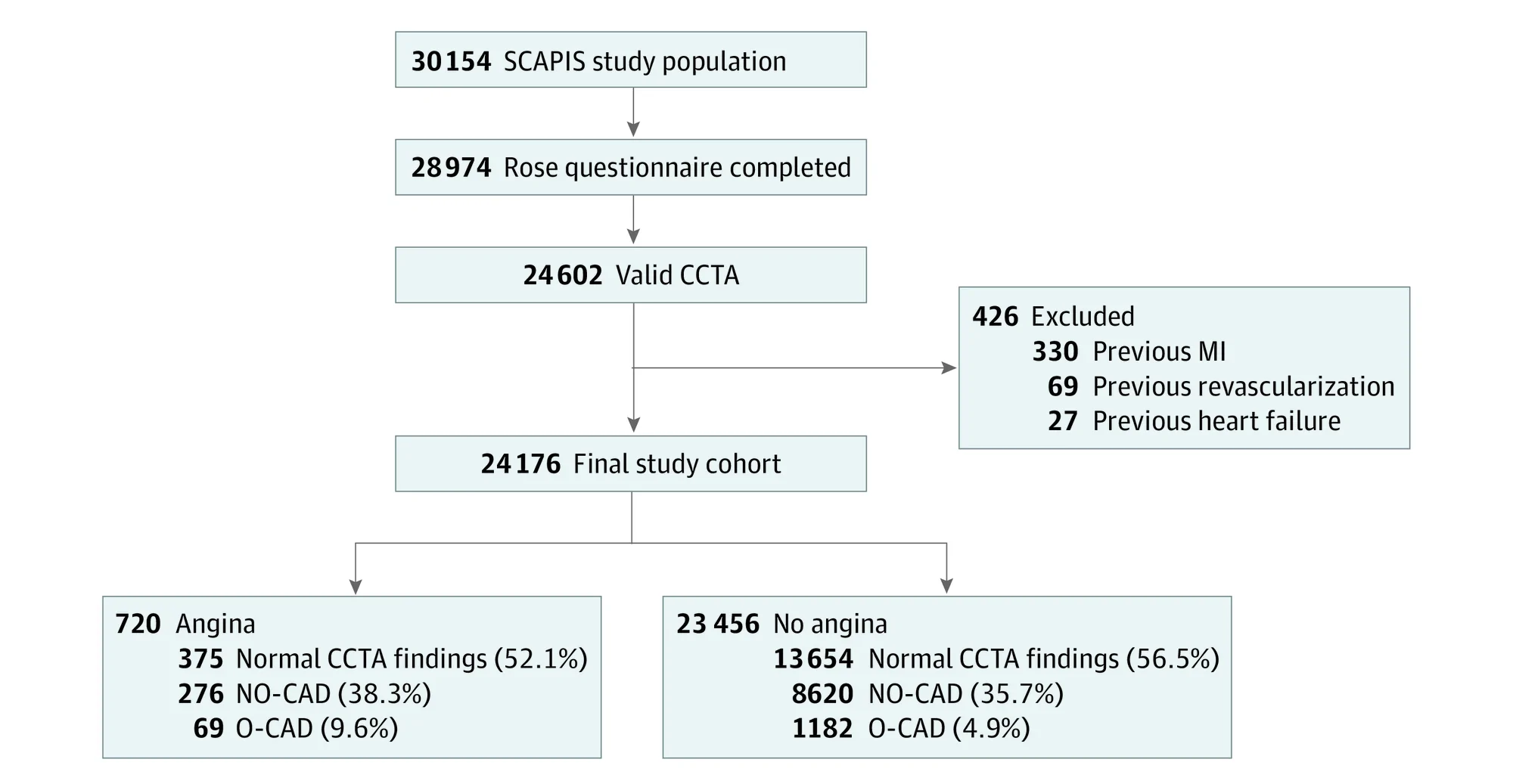
Study Population Flowchart CCTA indicates coronary computed tomography angiography; MI, myocardial infarction; NO-CAD, nonobstructive coronary artery disease; O-CAD, obstructive coronary artery disease; SCAPIS, Swedish Cardiopulmonary Bioimage Study.

According to the Rose Angina Questionnaire categorization, 720 participants (3.0%) in the study cohort had angina (515 [2.1%] with definite angina and 205 [0.8%] with probable angina). A majority of the study cohort (14 029 individuals [58.0%]) did not have any coronary atherosclerosis at CCTA. However, when coronary atherosclerosis was prevalent, it was significantly more common in those with angina than those without for obstructive CAD (69 individuals [9.6%] vs 1182 individuals [4.9%]; *P* < .001) and SIS greater than 4 (124 individuals [17.2%] vs 2749 individuals [11.7%]; *P* < .001) (eTable 4 in Supplement 1). Coronary atherosclerosis was also numerically more common in those with angina for nonobstructive CAD, but this finding was not significant (276 individuals [38.3%] vs 8620 individuals [35.7%]; *P* = .39).

Background variables stratified by angina status and CAD severity are presented in the Table. Compared with participants without angina, those with angina were older, more frequently female, and more likely to live alone. They were also more often born outside of Sweden and had lower educational and economic status. Active smoking, low physical activity, higher body mass index, and greater levels of stress and self-reported depression were more common among those with angina. Angina was further associated with higher prevalences of all comorbidities except for chronic kidney disease, which was rare in the cohort.

**Table.** Background Variables at SCAPIS Baseline According to Angina Status and CAD Category

Characteristic	Participants, No. (%) (N = 24 176)					
	No findings^a^		NO-CAD		O-CAD	
	Angina (n = 375)	No angina (n = 13 654)	Angina (n = 276)	No angina (n = 8620)	Angina (n = 69)	No angina (n = 1182)
Sociodemographic factors						
Age, median (IQR), y	57 (53-60)	56 (53-60)	60 (56-62)	59 (55-62)	60 (56-62)	60 (56-63)
Sex						
Female	273 (72.8)	8376 (61.3)	127 (46.0)	3185 (36.9)	18 (26.1)	246 (20.8)
Male	102 (27.2)	5278 (38.7)	149 (54.0)	5435 (63.1)	51 (73.9)	936 (79.2)
Civil status: single, divorced, or widowed	143 (38.5)	3392 (25.0)	92 (33.6)	2168 (25.3)	19 (27.9)	293 (25.0)
Born outside of Sweden	148 (39.6)	1832 (13.4)	108 (39.1)	1261 (14.6)	29 (42.0)	209 (17.7)
Highest level of education: elementary school	60 (16.0)	998 (7.3)	43 (15.8)	862 (10.0)	8 (11.8)	132 (11.2)
Economic status: low or unstable	31 (8.8)	272 (2.0)	33 (12.7)	191 (2.3)	5 (7.7)	32 (2.8)
Lifestyle factors						
Smoking status: current	61 (16.4)	1367 (10.1)	58 (21.2)	1238 (14.6)	14 (20.6)	207 (17.7)
Physical activity: never or low	69 (18.8)	862 (6.4)	59 (22.2)	754 (8.9)	12 (18.5)	111 (9.5)
Body mass index, mean (SD)^b^	29.0 (5.0)	26.3 (4.2)	29.3 (5.1)	27.3 (4.2)	28.4 (3.8)	27.8 (4.2)
Psychology						
High degree of general stress	153 (41.6)	2884 (21.3)	114 (42.7)	1576 (18.5)	24 (36.4)	185 (15.8)
Self-perceived depression	184 (50.8)	3716 (27.6)	140 (53.4)	2112 (25.0)	30 (46.9)	259 (22.2)
Comorbidities						
Hypertension	87 (23.8)	1702 (12.6)	94 (35.1)	2072 (24.3)	27 (39.7)	392 (33.6)
Hyperlipidemia	28 (7.6)	488 (3.6)	33 (12.3)	865 (10.2)	13 (19.1)	188 (16.1)
Diabetes	13 (3.5)	208 (1.5)	24 (8.9)	362 (4.3)	6 (8.8)	90 (7.7)
Atrial fibrillation or flutter	6 (1.6)	151 (1.1)	21 (7.7)	162 (1.9)	1 (1.5)	26 (2.2)
Chronic kidney disease	6 (1.6)	199 (1.5)	3 (1.1)	106 (1.2)	0 (0.0)	18 (1.5)
Obstructive sleep apnea syndrome	27 (7.4)	391 (2.9)	31 (11.4)	461 (5.4)	6 (8.8)	76 (6.5)
Chronic obstructive pulmonary disease	2 (0.5)	12 (0.1)	4 (1.4)	17 (0.2)	1 (1.4)	8 (0.7)
Previous stroke	0	4 (0.1)	1 (0.4)	14 (0.2)	2 (2.9)	11 (0.9)

Abbreviations: CAD, coronary artery disease; NO-CAD, nonobstructive CAD; O-CAD, obstructive CAD; SCAPIS, Swedish Cardiopulmonary Bioimage Study.

^a^ No findings refers to no findings on coronary computed tomography angiography.

^b^ Calculated as weight in kilograms divided by height in meters squared.

Among participants with angina, those with less severe CAD were younger, more frequently female, more often living alone, and less likely to be active smokers. Following an increase in CAD severity, participants with angina had an increasing prevalence of cardiovascular comorbidities and chronic respiratory obstructive disease. Medication use at SCAPIS baseline reflected the same pattern, with greater use of antihypertensives and lipid-lowering therapy among participants with angina than among those without, and with increasing use across CAD severity categories (eTable 5 in Supplement 1).

### Outcomes

During a median (IQR) follow-up time of 7.7 (6.9-8.6) years, 293 participants experienced the primary end point (MI or coronary death), including 21 events among those with angina and 272 events among those without (cumulative incidence, 2.9% vs 1.2%; HR, 2.50; 95% CI, 1.60-3.90; *P* < .001) (Figure 2A and Figure 3A). Event rates increased with CAD severity (Figure 2B), with no significant interaction between angina and degree of CAD (*P* for interaction = .65). After adjustment for possible confounders, the hazard of the primary end point was attenuated in the partial adjustment model (HR, 1.78; 95% CI, 1.12-2.83), with minimal further attenuation in the full adjustment model including CAD severity category and SIS (HR, 1.65; 95% CI, 1.04-2.63). There were still no signs of interaction between angina and degree of CAD in the partially adjusted model (*P* for interaction = .79) and the fully adjusted model (*P* for interaction = .90).

**Figure 2.**
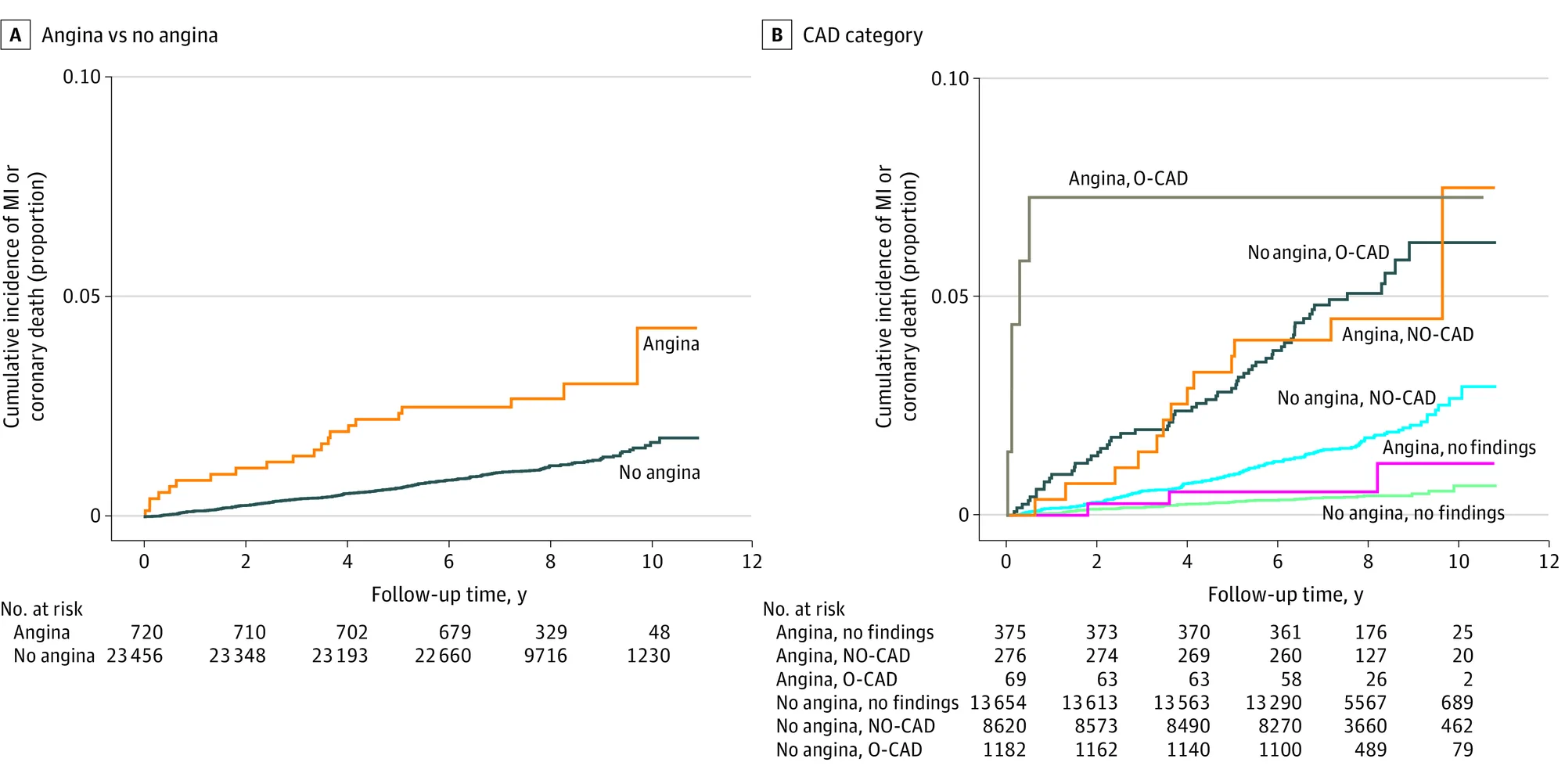
Kaplan-Meier Curves for the Primary End Point of Myocardial Infarction (MI) or Coronary Death Cumulative incidence of the primary end point (MI or coronary death) in all participants with angina compared with no angina (A) and stratified by coronary artery disease (CAD) category (B). No findings refers to no findings on coronary computed tomography angiography. NO-CAD indicates nonobstructive CAD; O-CAD, obstructive CAD.

**Figure 3.**
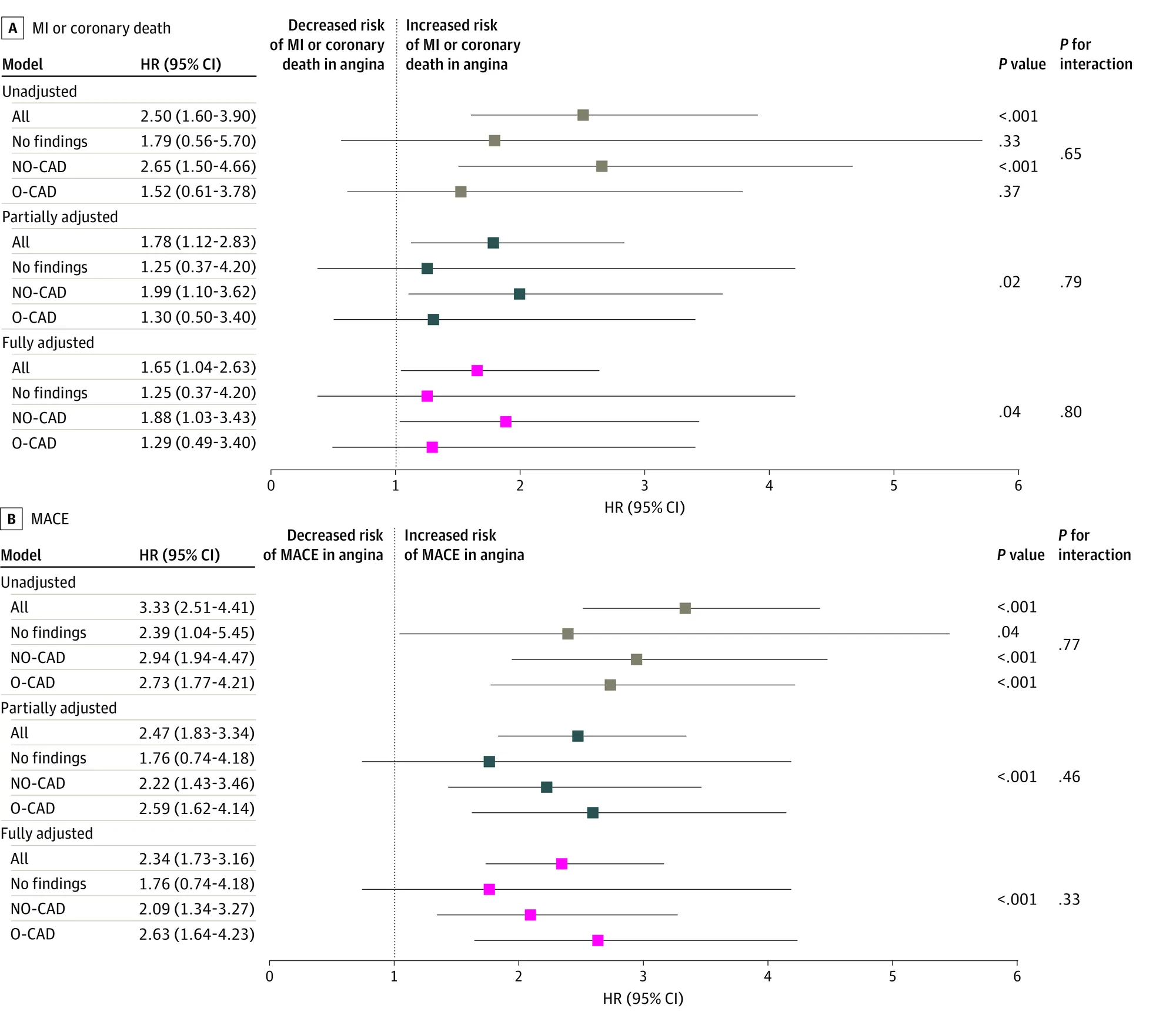
Dot Plot of Cox Regression Results for Univariable and 2-Step Multivariable Adjustment Models Risk estimates (hazard ratios [HRs]) for the primary (myocardial infarction [MI] or coronary death) (A) and secondary (major adverse cardiovascular event [MACE]) (B) end points. No findings refers to no findings on coronary computed tomography angiography. NO-CAD indicates nonobstructive coronary artery disease; O-CAD, obstructive coronary artery disease.

For the secondary MACE outcome, 584 events occurred overall, including 53 events (7.4%) among participants with angina and 531 events (2.3%) among participants with no angina (HR, 3.33; 95% CI, 2.51-4.41; *P* < .001), also with increasing event rates by CAD severity (Figure 3B and Figure 4A-B). Multivariable adjustment attenuated the hazard of the secondary end point in the partially adjusted model (HR, 2.47; 95% CI, 1.83-3.34), and further in the fully adjusted model (HR, 2.34; 95% CI, 1.73-3.16). Interaction analyses were nonsignificant in the unadjusted (P for interaction = .77), partially adjusted (*P *for interaction = .46), and fully adjusted (*P *for interaction = .33) models. The 2 sensitivity analysis models did not change the estimated HRs for either the primary or secondary end point (eTable 6 in Supplement 1).

**Figure 4.**
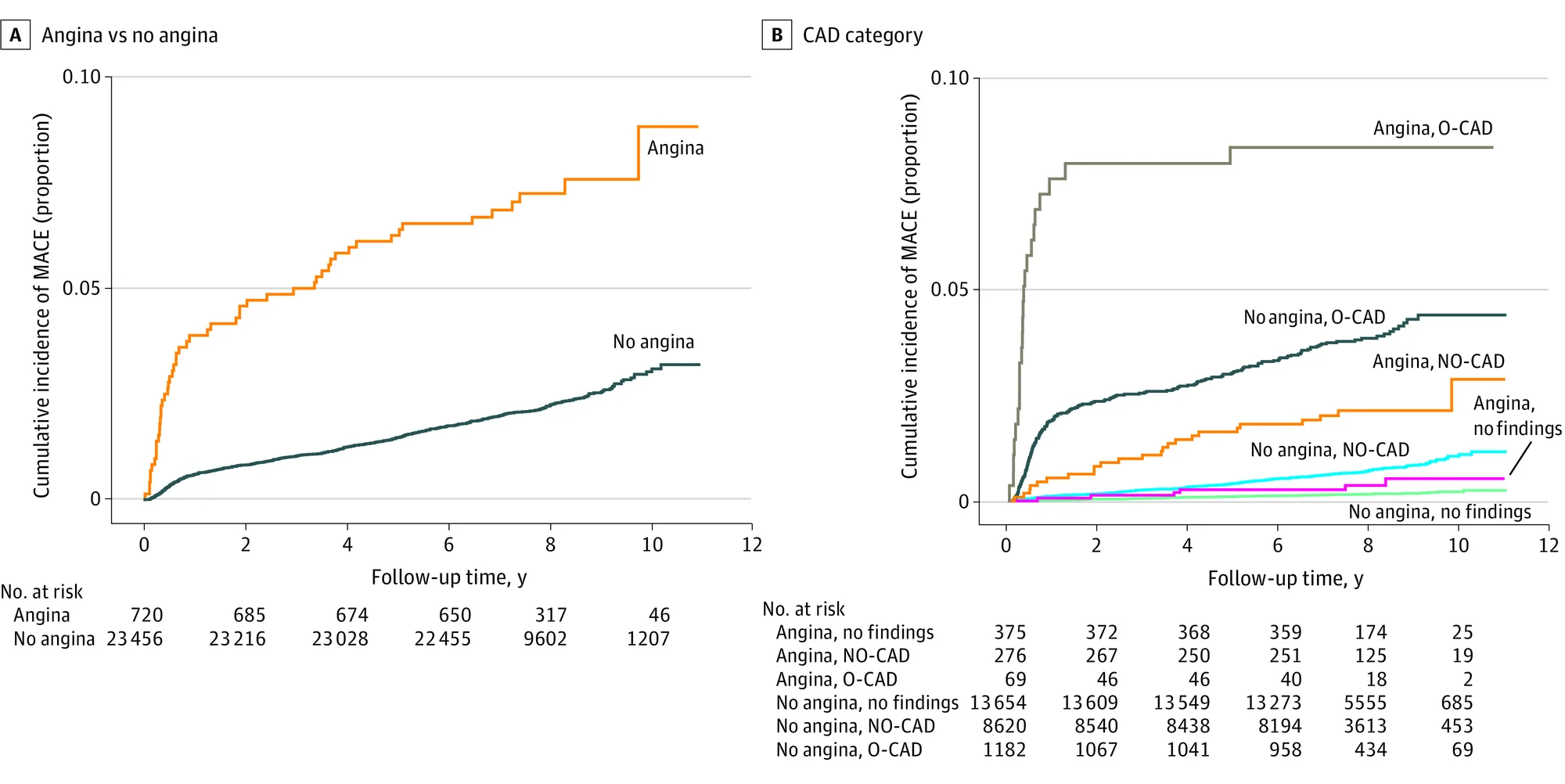
Kaplan-Meier Curves for the Secondary End Point of Major Adverse Cardiovascular Events (MACE) Cumulative incidence of MACE in all participants with angina compared with no angina (A) and stratified by coronary artery disease (CAD) category (B). No findings refers to no findings on coronary computed tomography angiography. NO-CAD indicates nonobstructive coronary artery disease; O-CAD, obstructive coronary artery disease.

The tertiary heart failure end point occurred in 68 participants overall, including 6 participants (0.8%) with angina and 62 participants (0.3%) with no angina (HR, 3.07; 95% CI, 1.33-7.01; *P* = .009). Adjustment for age and sex (HR, 3.19; 95% CI, 1.38-7.39), and additionally for CAD and SIS (HR, 2.95; 95% CI, 1.27-6.84), did not affect the results.

## Discussion

In this large contemporary general population–based cohort of randomly selected middle-aged Swedish women and men without previous MI, coronary revascularization, or heart failure, we evaluated the long-term significance of angina pectoris in association with CAD severity. The principal findings were (1) the presence of angina was associated with worse outcomes across all categories of CAD; (2) the observed risk was partly attributable to sociodemographic, medical, and lifestyle factors, whereas plaque burden appeared to exert only a modest additional effect; and (3) even after multivariable adjustment, angina remained associated with a significantly increased risk of MI or coronary death, suggesting that other pathophysiological mechanisms such as CMD or coronary vasomotor disorders may contribute.

This study uniquely allowed assessment of coronary pathology in individuals with and without angina, providing insights into the interplay between symptoms and CAD. Consistent with previous studies, event rates of MI and coronary death increased with greater CAD severity.^13,14^ These gradients were evident across the entire cohort but remained higher in angina; individuals with angina and nonobstructive CAD had similar event rates of MI and coronary death as those with asymptomatic obstructive CAD. Among individuals with angina and obstructive CAD, most events occurred early during follow-up. Prodromal angina is common among patients experiencing a first MI,^27,28^ but increased symptom awareness and care-seeking behavior following study participation may also contribute. Regarding the secondary MACE outcome, similar event rate patterns were observed. In some events, this may reflect study-related revascularization because SCAPIS participants with severe obstructive CAD findings on CCTA were eligible for clinical referral.

To disentangle the contributions of clinical background factors and CCTA-derived measures to the association of angina pectoris with outcomes, we applied 2 sequential adjustment models. Adjustment for sociodemographic factors, lifestyle variables, and comorbidities resulted in partial attenuation. Addition of CAD severity and plaque burden was intended to evaluate whether the remaining association of angina could be explained by epicardial coronary atherosclerosis. The association remained largely unchanged and, although plaque burden has prognostic importance,^12,15,16^ these findings suggest that other mechanisms may contribute. CMD is a recognized determinant of angina pectoris and has been independently associated with MACE among patients with angina and nonobstructive CAD.^1,29,30,31,32^ In selected cohorts of patients with myocardial infarction and nonobstructive coronary arteries undergoing coronary vasomotor testing with acetylcholine, epicardial or microvascular vasospasm has been identified in half of patients.^33,34,35^ Although not assessed in this study, CMD or vasomotor disorders may partly explain the remaining association of angina with adverse cardiac outcomes.

To our knowledge, no previous population-based study has evaluated the prognostic significance of angina in association with CAD. In a Danish angiography cohort, patients with angina and nonobstructive CAD exhibited increased long-term risk of MACE compared with asymptomatic controls, although the prognostic importance of angina was not directly assessed.^17^ Previous Rose Angina Questionnaire cohorts have demonstrated adverse long-term outcomes among individuals with angina compared with asymptomatic controls, but lacked contemporary coronary imaging data.^5,6,7,8^

Although the Rose Angina Questionnaire is designed primarily for epidemiological use, our findings suggest that symptom-based assessment remains relevant when evaluating cardiovascular risk. In the current imaging era, the observed associations indicate that angina symptoms may provide information complementary to clinical background factors and coronary imaging findings.

### Strengths and Limitations

The major strengths of this study include the large, randomly selected population-based cohort drawn from across Sweden and the high quality and low degree of missingness in SCAPIS CCTA and outcome data. Previous evaluations suggest only modest selection bias within SCAPIS, supporting generalizability of the findings.^36^ Further, cardiovascular disease statistics report similar incidence and prevalence of ischemic heart disease in Sweden compared with other high-income countries in Europe and the US.^37,38^

This study also has limitations. Self-reporting of sociodemographic, lifestyle, and medical history information constitutes a limitation due to the risk of misclassification bias, although likely nondifferential and of limited impact on results. We lacked detailed information on medical treatments, as well as data on participants’ care-seeking patterns. Such information could have provided additional context for interpreting the association of angina pectoris with clinical outcomes. However, medication use likely reflects underlying disease burden and clinical management and should primarily be interpreted as part of the disease pathway rather than as confounding factors. The Rose Angina Questionnaire is well validated and demonstrates good reproducibility among both women and men.^9,39^ A potential limitation is that in SCAPIS, the original site-map question concerning chest pain location was omitted for technical reasons, which could introduce misclassification. However, previous studies suggest that the prognostic information captured by the Rose Angina Questionnaire is largely driven by exertional symptoms and is preserved in abbreviated versions.^24,25,40^ Because the questionnaire only captures exertional symptoms, participants with chest pain occurring exclusively at rest, such as some individuals with vasospastic angina, may have been misclassified as not having angina. Information bias in outcome definitions represents another potential limitation, particularly for heart failure hospitalization because *ICD-10* coding restricted to primary diagnoses may miss clinically relevant secondary diagnoses. A potential statistical limitation is that the lower number of events in some subgroup analyses may have reduced the precision of the adjusted risk estimates; these analyses should therefore be interpreted primarily as an assessment of consistency across CAD categories. However, the total number of events in the main analyses was sufficient for multivariable adjustment.

## Conclusion

In this cohort study of a large contemporary cohort of randomly selected, middle-aged women and men from the general population, angina pectoris was independently associated with an increased long-term risk of MI or coronary death, irrespective of CAD severity on CCTA. Although clinical background factors accounted for part of the observed association, plaque burden and stenosis severity provided only limited additional explanatory value. The persistent association suggests that alternative pathophysiological mechanisms, including CMD and vasomotor disorders, may have contributed to the excess risk.

These findings indicate that symptom-based assessment remains relevant in the modern imaging era. Further studies are warranted to clarify the underlying mechanisms and to determine whether functional coronary disorders contribute to the observed associations.

## Notes

Supplement 1. eTable 1. ICD-10 codes and KVA-codes used for exclusion criteria and outcome components eFigure 1. Rose angina questionnaire categorization eFigure 2. Directed acyclic graph regarding the primary endpoint (A) and secondary endpoint (B) eTable 2. Missing data on background variables at SCAPIS baseline eTable 3. Study population background variables at SCAPIS baseline examination, including assessment/measurement method in SCAPIS eTable 4. Segment involvement score (SIS) distribution in individuals with and without angina eTable 5. Medical treatment at SCAPIS baseline, stratified by angina status and CAD severity category (N=24,176) eTable 6. Sensitivity analyses: prevalence of related variables and multivariable Cox regression estimates for the sensitivity analysis models 1 (adding self-reported previous MI, revascularization or heart failure) and 2 (also adding presence of aortic valve calcification at CCTA)

Supplement 2. Data sharing statement
