# Supplementary material for: Importance of Angina Pectoris by Coronary Artery Disease Severity
Source: JAMA Netw Open. 2026 Nov 11;9(11):e2643292. doi: 10.1001/jamanetworkopen.2026.43292 (PMC13756133; doi:10.1001/jamanetworkopen.2026.43292)
Supplement: Supplementary file 1 [file jamanetwopen-e2643292-s001.pdf]

Supplemental Online Content

Welén Schef K, Dworeck C, Johnston N, et al. Importance of angina pectoris by coronary artery disease severity. *JAMA Netw Open*.2026;9(11):e2643292.
doi:10.1001/jamanetworkopen.2026.43292

eTable 1. *ICD-10* codes and KVA-codes used for exclusion criteria and outcome components
eFigure 1. Rose angina questionnaire categorization
eFigure 2. Directed acyclic graph regarding the primary endpoint (A) and secondary endpoint (B)
eTable 2. Missing data on background variables at SCAPIS baseline
eTable3. Study population background variables at SCAPIS baseline examination, including assessment/measurement method in SCAPIS
eTable 4. Segment involvement score (SIS) distribution in individuals with and without angina
eTable 5. Medical treatment at SCAPIS baseline, stratified by angina status and CAD severity category (N=24,176)
eTable 6. Sensitivity analyses: prevalence of related variables and multivariable Cox regression estimates for the sensitivity analysis models 1 (adding self-reported previous MI, revascularization or heart failure) and 2 (also adding presence of aortic valve calcification at CCTA)

This supplemental material has been provided by the authors to give readers additional information about their work.

© 2026 Welén Schef K et al. *JAMA Network Open*.

eTable 1. *ICD-10* codes and KVA-codes used for exclusion criteria and outcome components.

	National Patient Registry ICD-10 codes, in main position	National Patient Registry KVA-codes	National Cause of Death Register ICD10-codes, in main position
**Exclusion criteria**			
Myocardial infarction	I21, I22 (prior to CCTA)		
Revascularization		FNA, FNB, FNC, FND, FNE, FNF, FNG (prior to CCTA)	
Heart failure	I50 (prior to CCTA)		
**Outcome components**			
Myocardial infarction	I21, I22		
Revascularization	I20-25	FNA, FNB, FNC, FND, FNE, FNF, FNG	
Heart failure	I50		
Coronary death			I20, I21, I22, I23, I24, I25
Heart failure death			I50

© 2026 Welén Schef K et al. *JAMA Network Open*.

eFigure 1. Rose Angina Questionnaire categorization.

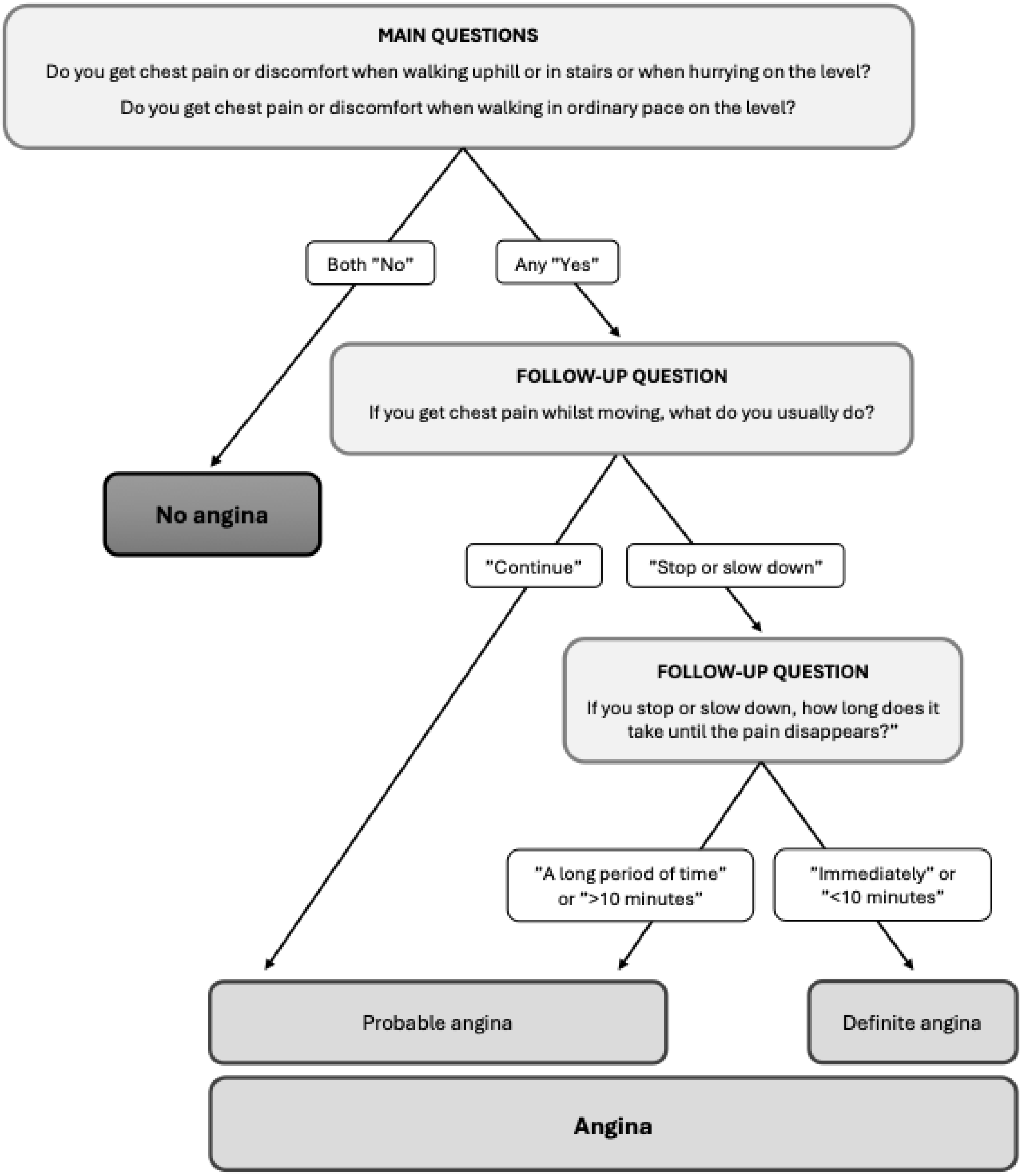

© 2026 Welén Schef K et al. *JAMA Network Open*.

eFigure 2. Directed acyclic graph regarding the primary endpoint (A) and secondary endpoint (B).

A.

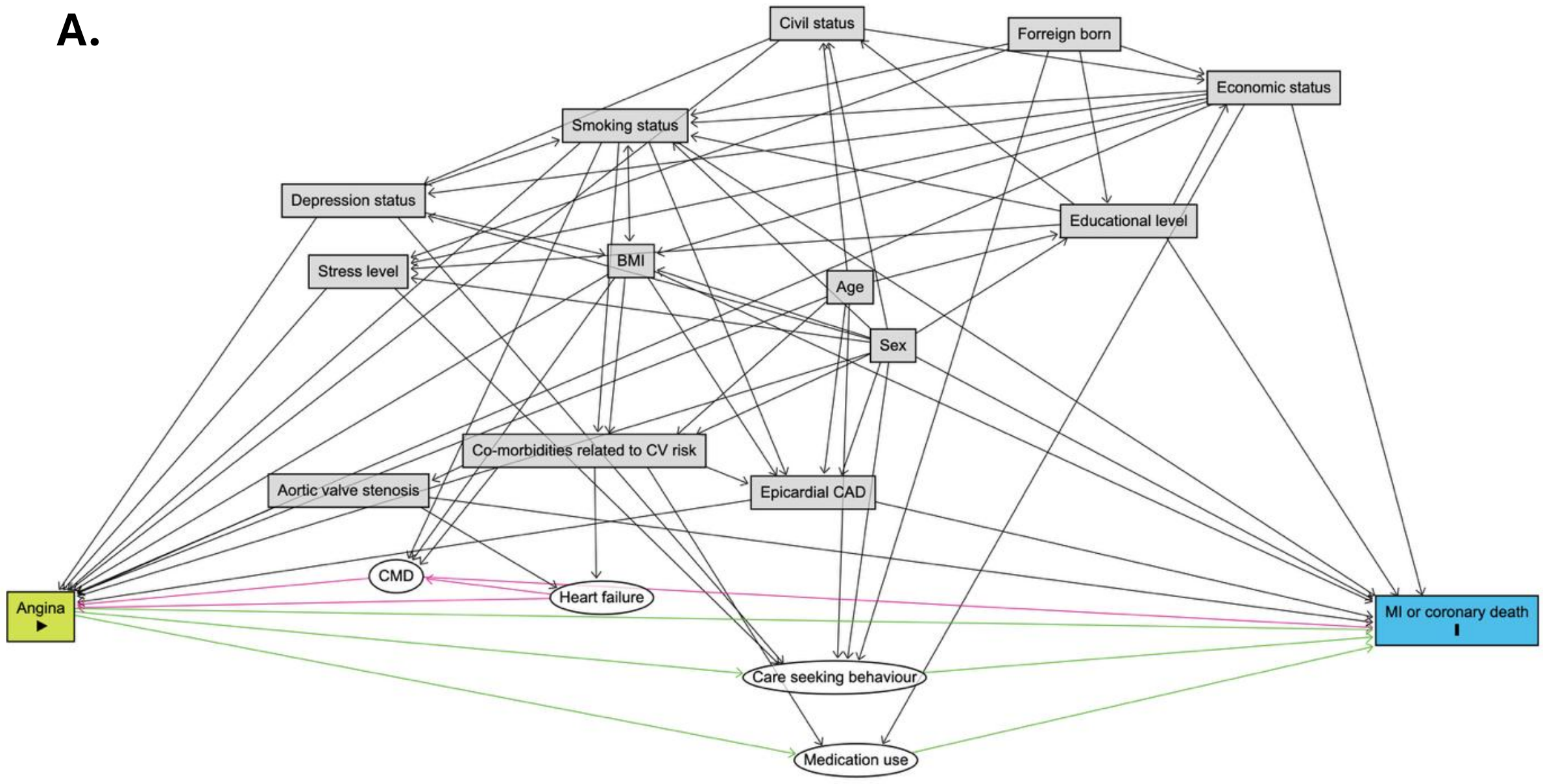

B.

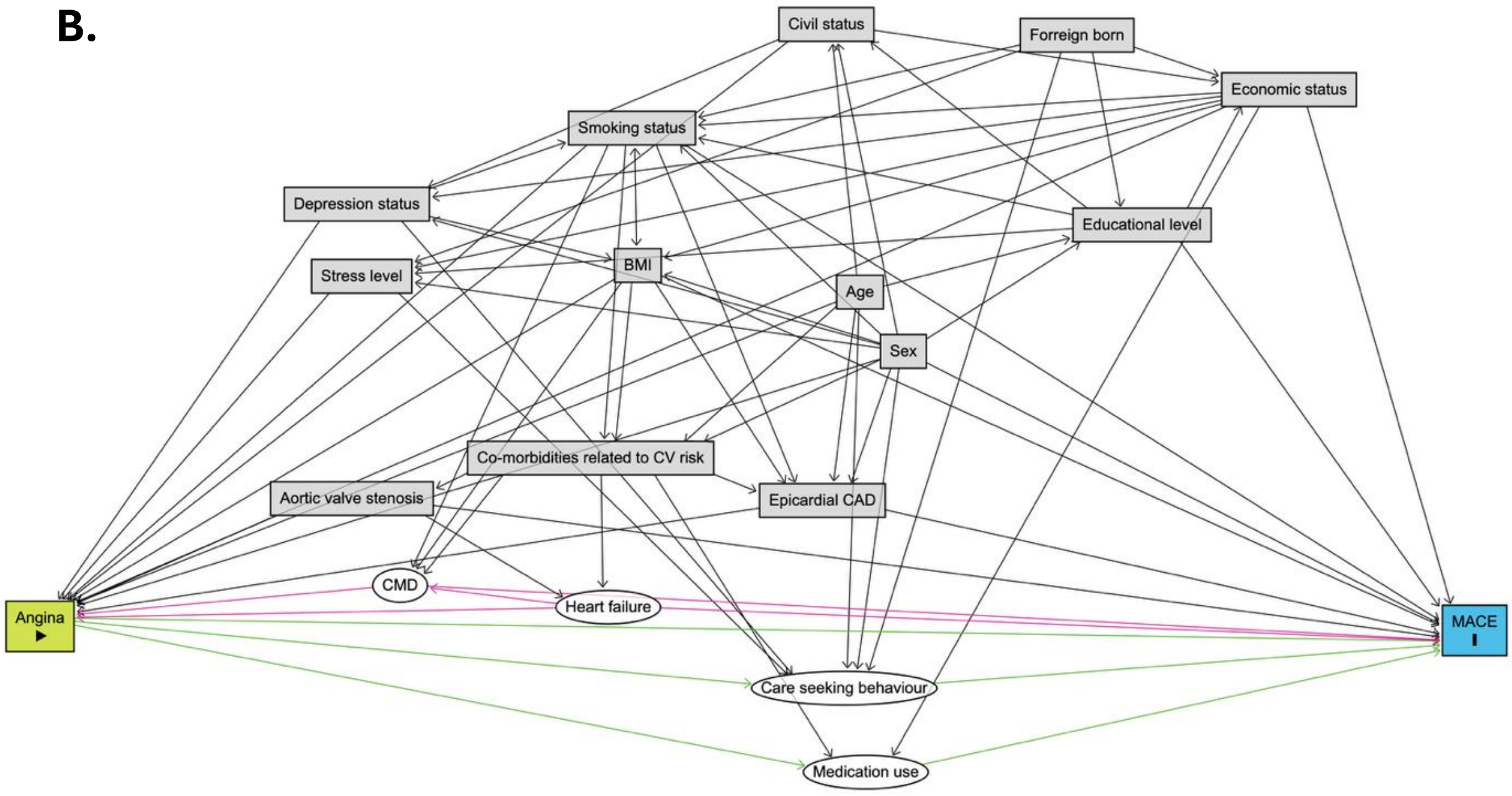

Green box: exposure
Blue box: outcome
Grey boxes: factors with available data
White circles: factors with unavailable data
Grey arrows: confounding paths, correctly closed by adjustment
Red arrows: confounding paths, open (residual confounding)
Green arrows: mediating paths, open

© 2026 Welén Schef K et al. *JAMA Network Open*.

eTable 2. Missing data on background variables at SCAPIS baseline.

	Missing values
	N=24,176
**Background variables**	
Age	0
Female	0
Male	0
Civil status - Single/Divorced/Widowed	133 (0.6)
Born outside of Sweden	11 (0.1)
Education, highest level - Elementary school	66 (0.3)
Economic status - Low/Unstable	508 (2.1)
Smoking status - Current	307 (1.3)
Physical activity - Never/Low	237 (1.0)
BMI (mean/SD)	0
High degree of general stress	257 (1.1)
Self-perceived depression	399 (1.7)
Hypertension	293 (1.2)
Hyperlipidemia	295 (1.2)
Diabetes Mellitus	291 (1.2)
Atrial fibrillation/flutter	284 (1.2)
Chronic kidney disease	17 (0.1)
OSAS	284 (1.2)
COPD	3 (0.1)
Previous stroke	3 (0.1)
**Multivariable adjustment models**	
Primary	1757 (7.3)
Secondary	1757 (7.3)
Tertiary	5 (0.1)

Data are presented as n (%)

© 2026 Welén Schef K et al. *JAMA Network Open*.

eTable3. Study population background variables at SCAPIS baseline examination, including assessment/measurement method in SCAPIS.

	**All** *N=24,176*	**Variable definition**
**Sociodemographic factors**		
Age *(median/IQR)*	57 (54-61)	N/A
Female	12,225 (50.6)	N/A
Male	11,951(49.4)	N/A
Civil status - Single/Divorced/Widowed	6,107 (25.4)	Self-reported in SCAPIS Questionnaire, as single/divorced/widowed or married/co-living
Born outside of Sweden	3,587 (14.8)	Self-reported in SCAPIS Questionnaire
Education, highest level - Elementary school	2,103 (8.7)	Self-reported in SCAPIS Questionnaire, as elementary school, high school or college/university
Economic status - Low/Unstable	564 (2.4)	Self-reported in SCAPIS Questionnaire, combined variable based on two questions on economic capacity, as low/unstable, intermediate or high/stable.
**Lifestyle factors**		
Smoking status - Current	2,945 (12.3)	Self-reported in SCAPIS Questionnaire as previous, current or never smoked.
Physical activity – Never/Low	1,867 (7.8)	Self-reported in SCAPIS Questionnaire, combined variable based on two questions on physical activity intensity and frequency (Grimby-scale)
BMI (mean/SD)	29.0 (5.0)	SCAPIS baseline examination measurement
**Psychology**		
High degree of general stress	4,936 (20.6)	Self-reported in SCAPIS Questionnaire, based on response to “Have you experienced a period of stress at work or at home within the last five years” used in the INTERHEART study
Self-perceived depression	6,441 (27.1)	Self-reported in SCAPIS Questionnaire, evaluated based on response to “Feeling sad, gloomy or depressed during a two-week period (or longer) in the last twelve months” and categorized as yes or no
**Co-morbidities**		
Hypertension	4,374 (18.3)	Self-reported in SCAPIS Questionnaire
Hyperlipidemia	1,615 (6.8)	Self-reported in SCAPIS Questionnaire
Diabetes Mellitus	703 (2.9)	Self-reported in SCAPIS Questionnaire
Atrial fibrillation/flutter	367 (1.5)	Self-reported in SCAPIS Questionnaire
Chronic kidney disease	332 (1.4)	Estimated glomerular filtration <60 ml/min/1.73m^2 by the CKD-EPI formula, based on SCAPIS baseline creatinine
OSAS	992 (4.2)	Self-reported in SCAPIS Questionnaire
COPD	44 (0.2)	Self-reported in SCAPIS Questionnaire
Previous stroke	32 (0.1)	Self-reported in SCAPIS Questionnaire

Data are presented as n (%).

© 2026 Welén Schef K et al. *JAMA Network Open*.

eTable 4. Segment involvement score (SIS) distribution in individuals with and without angina.

	Angina	No angina
	n=720	*n=23,456*
**SIS group**		
0	375 (52.1)	13,654 (58.2)
1	104 (14.4)	3,620 (15.4)
2-3	117 (16.3)	3433 (14.6)
>4	124 (17.2)	2749 (11.7)

Data are presented as n (%).

eTable 5. Medical treatment at SCAPIS baseline, stratified by angina status and CAD severity category (N=24,176).

	O-CAD		NO-CAD		No findings	
	Angina	No angina	Angina	No angina	Angina	No angina
	n=69	*n=1,182*	*n=276*	*n=8,620*	*n=375*	*n=13,654*
**Medical treatment**						
Anti-platelet	0 (0)	58 (4.9)	9 (3.3)	270 (3.1)	11 (2.9)	133 (1.0)
Lipid-lowering	20 (29.0)	304 (25.7)	68 (24.6)	1,666 (19.3)	59 (15.7)	1,382 (10.1)
Anti-hypertensive	3 (4.3)	42 (3.6)	14 (5.1)	180 (2.1)	11 (2.9)	121 (0.9)
Anti-diabetic	1 (1.4)	25 (2.1)	20 (7.2)	111 (1.3)	6 (1.6)	87 (0.6)

Data are presented as n (%).
Medical treatment defined as a filled prescription within 6 months prior to the baseline examination at SCAPIS index and confirmed having taken such medication during the last 2 weeks in the SCAPIS questionnaire.

© 2026 Welén Schef K et al. *JAMA Network Open*.

eTable 6. Sensitivity analyses: prevalence of related variables and multivariable Cox regression estimates for the sensitivity analysis models 1 (adding self-reported previous MI, revascularization or heart failure) and 2 (also adding presence of aortic valve calcification at CCTA).

	All	Angina	No angina	HR	HR in main analysis
	N=24,176	*n=720*	*n=23,456*		
**Related variables**					
Previous myocardial infarction, n (%)	1,316 (5.4)	55 (7.6)	1,261 (5.4)		
Previous revascularization, n (%)	69 (0.3)	12 (1.7)	57 (0.2)		
Previous heart failure, n (%)	109 (0.5)	18 (2.5)	91 (0.4)		
Aortic valve calcification, n (%)	1,606 (6.7)	65 (9.1)	1,541 (6.6)		
**Sensitivity analysis 1**					
Primary outcome					
Model 1 – partially adjusted				1.80	1.78
Model 2 – fully adjusted				1.69	1.65
Secondary outcome					
Model 1 – partially adjusted				2.42	2.47
Model 2 – fully adjusted				2.34	2.34
Tertiary outcome					
Model 1 – age- and sex				1.87	3.19
Model 2 – age, sex, CAD and SIS				1.78	2.95
**Sensitivity analysis 2**					
Primary outcome					
Model 1 – partially adjusted				1.79	1.78
Model 2 – fully adjusted				1.69	1.65
Secondary outcome					
Model 1 – partially adjusted				2.29	2.47
Model 2 – fully adjusted				2.22	2.34
Tertiary outcome					
Model 1 – age- and sex				1.11	3.19
Model 2 – age, sex, CAD and SIS				1.10	2.95

© 2026 Welén Schef K et al. *JAMA Network Open*.
