# Supplementary material for: Importance of Angina Pectoris by Coronary Artery Disease Severity
Source: JAMA Netw Open. 2026 Nov 11;9(11):e2643292. doi: 10.1001/jamanetworkopen.2026.43292 (PMC13756133; doi:10.1001/jamanetworkopen.2026.43292)
Supplement: Supplementary file 2 [file jamanetwopen-e2643292-s002.pdf]

Data Sharing Statement

Welén Schef. Prognostic Significance of Angina Pectoris for Assessing Coronary Artery Disease Severity. *JAMA Netw Open*. Published November 11, 2026.
doi:10.1001/jamanetworkopen.2026.43292

Data
Data available: No

Additional Information
Explanation for why data not available: Due to the nature of the sensitive personal data and study materials they cannot be made freely available. However, by contacting the corresponding author or study organization (www.scapis.org), procedures for sharing data, analytic methods, and study materials for reproducing the results or replicating the procedure can be arranged following Swedish legislation. The corresponing author Kerstin Welén Schef had full access to all the data in the study and takes responsibility for the integrity of the data and the accuracy of the data analysis.
